# Enhancing performance of ZnO dye-sensitized solar cells by incorporation of multiwalled carbon nanotubes

**DOI:** 10.1186/1556-276X-7-166

**Published:** 2012-03-05

**Authors:** Wei-Chen Chang, Yao-Yi Cheng, Wan-Chin Yu, Yih-Chun Yao, Chia-Hua Lee, Hung-Han Ko

**Affiliations:** 1 Institute of Organic and Polymeric Materials, National Taipei University of Technology, Taipei, Taiwan, 10608, Republic of China; 2 Green Energy and Environment Research Laboratories, Industrial Technology Research Institute, Hsinchu, Taiwan, 31053, Republic of China; 3 Department of Molecular Science Engineering, National Taipei University of Technology, Taipei, Taiwan, 10608, Republic of China

**Keywords:** ZnO, nanoparticle, multiwalled carbon nanotube, composite film, dye-sensitized solar cells, conversion efficiency

## Abstract

A low-temperature, direct blending procedure was used to prepare composite films consisting of zinc oxide [ZnO] nanoparticles and multiwalled carbon nanotubes [MWNTs]. The mesoporous ZnO/MWNT films were fabricated into the working electrodes of dye-sensitized solar cells [DSSCs]. The pristine MWNTs were modified by an air oxidation or a mixed acid oxidation treatment before use. The mixed acid treatment resulted in the disentanglement of MWNTs and facilitated the dispersion of MWNTs in the ZnO matrix. The effects of surface property and loading of MWNTs on DSSC performance were investigated. The performance of DSSCs was found to depend greatly on the type and the amount of MWNTs incorporated. At a loading of 0.01 wt%, the acid-treated MWNTs were able to increase the power conversion efficiency of fabricated cells from 2.11% (without MWNTs) to 2.70%.

## Introduction

Dye-sensitized solar cells [DSSCs] are considered as promising third-generation solar energy devices because of their low fabrication cost, compatibility with flexible substrates, and practicable high conversion efficiency [1,2]. The maximum conversion efficiencies attained by DSSCs so far (approximately 11%), although considerably lower than those of silicon solar cells, may meet the requirements of many practical applications [1,3]. Further improvement in the conversion efficiency of DSSCs is possible, as theoretical prediction of the maximum conversion efficiency of DSSCs is approximately 20% [4].

A DSSC is a photoelectrochemical system in which a porous nanostructured oxide film with adsorbed dye molecules acts as the photoanode and plays a significant role in converting photons into electrical energy. It has been shown that the performance of DSSCs is closely related to the structure of the photoelectrode film [5]. A rational design of the photoelectrode film structure may result in better light harvesting and electron transport. Zinc oxide [ZnO] has been used widely for the fabrication DSSC photoanodes. ZnO is regarded as an attractive alternative to titanium dioxide [TiO_2_] because it has a similar band gap level to that of TiO_2 _while possesses a higher electron mobility and more flexibility in synthesis and morphologies [6,7]. Among the nanostructures investigated for the fabrication of DSSC photoelectrode films, nanoparticles are most widely used. This is likely due to a high specific surface area provided by nanoparticle-based films. Another reason is probably the ease of preparation of nanoparticles through simple chemical solution methods and the ease of film formation through the doctor-blade method.

Nanoparticle-based films can provide a large surface area, but the existence of numerous boundaries in the nanoparticle network may hinder the transport of electrons in photoelectrode and thus limit energy conversion efficiency of DSSCs. Incorporating one-dimensional [1-D] nanostructures into nanoparticulate films may overcome the problem by providing direct pathways for electron transport [8]. Carbon nanotubes [CNTs], a type of 1-D nanostructure, possess several unique properties including hollow and layered structures, a high aspect ratio, excellent electrical and thermal conductivity, high mechanical strength, and a large specific surface area [9]. Incorporating CNTs into ZnO electrodes should provide highly conductive paths to the ZnO nanostructures, thereby promoting faster transport of photo-induced electrons in DSSCs and higher current. The combination of CNTs with ZnO nanoparticles is thus a promising approach to boost conversion efficiency of DSSCs. In fact, this approach has been used to improve the performance of TiO_2_-based DSSCs [10-12]. However, the effect of CNTs on ZnO nanoparticle-based DSSCs has not been reported before. The major barrier for the application of CNTs in DSSCs is the insolubility of CNTs in most solvents. To obtain homogeneous dispersion of CNTs, CNTs need to be pre-treated before mixing them with nanoparticles. The air oxidation treatment has been found to completely remove amorphous carbon and metal oxide impurities from CNTs [13]. The mixed acid treatment not only effectively purifies CNTs but also leads to the formation of oxygen-containing groups, mainly carboxylic, on the graphitic surface [14,15]. The carboxylic groups thus formed facilitate the exfoliation of CNT bundles and therefore the dispersion of CNTs. The oxygen-containing groups should also improve the interfacial bonding between CNTs and ZnO nanoparticles.

In this study, DSSC photoanodes were fabricated using nanostructured films based on commercial ZnO nanoparticles. To study the effects of incorporating CNTs on device performance, two different types of multiwalled carbon nanotubes [MWNTs], i.e., oxidized MWNT [O-MWNT] (or oxidized in air) and acid-MWNT (mixed acid treated), were prepared and blended with ZnO nanoparticles at various levels. The effects of air oxidation and mixed acid treatments on the morphologies of MWNT were studied using transmission electron microscopy [TEM], and the morphologies of the prepared ZnO/MWNT composite films were investigated by using field emission scanning electron microscopy [FE-SEM]. The energy conversion efficiency [*η*] and the electrochemical impedance of the fabricated DSSCs were also determined.

## Experimental methods

Pristine MWNTs that were 10 to 30 nm in diameter and 5 to 15 mm in length were purchased from Nanotech Port (Genesis Nanotech Corporation, Tainan, Taiwan, Republic of China). These MWNTs were produced via a chemical vapor deposition method. To obtain the O-MWNT, the pristine unmodified-MWNT [U-MWNT] was purified by thermal treatment in air at 550°C for 45 min to remove amorphous carbon and residual metal catalysts. The carboxylic group-modified MWNT [acid-MWNT] was prepared by treating the O-MWNT with a sulfuric acid/nitric acid mixture (3:1, *v/v*) at 50°C for 2 h under ultrasonication. After the mixed acid treatment, the acid-MWNT was collected by filtration and then washed with distilled water and methanol [16].

Commercial ZnO nanoparticles (UniRegion Bio-Tech, Taiwan, Republic of China) that were about 20 nm in size were dispersed in equal proportion of distilled water and *tert*-butanol to form ZnO pastes. The MWNTs were added at this step when ZnO/MWNT composite films were to be made. The nanoporous films were prepared by applying the pastes onto commercial fluorine-doped tin oxide [FTO] substrates (Nippon Sheet Glass, 8 to 10 Ω/□, 3 mm thick) (Nippon Sheet Glass Company, Tokyo, Japan) by the doctor-blade method using adhesive tapes as a frame and spacer. The active electrode area was 0.25 cm^2^, and the films had a thickness of 8 μm. The resulting films were then gradually heated and annealed at 150°C for 1 h to remove organic materials in the paste and to increase crystallinity of ZnO. After being cooled to room temperature, the porous films were sensitized by immersing them into a dye solution that contained 0.5 mM of cis-bis (isothiocyanato) bis-(2,2'-bipyridyl-4,4'-dicarboxylato)-ruthenium(II)bis-tetrabutylammonium (Solaronix, N719) (Solaronix SA, Aubonne, Switzerland) in a mixed solvent consisting of equal parts of acetonitrile and *tert*-butanol. The dye-adsorption time lasted for 2 h. The dye-loaded electrodes were then rinsed with acetonitrile and dried in the air. The counter electrode was made of FTO glass, onto which a nanocrystalline platinum [Pt] catalyst was deposited by decomposition of H_2_PtCl_6 _at 400°C for 20 min. The ZnO photoanode and the counter electrode were sealed together with a 60 μm-thick hot-melting spacer (DuPont, Surlyn, Shanghai, People's Republic of China), and the inner space was filled through a hole with a volatile electrolyte composed of 0.1 M lithium iodide, 0.6 M 1, 2-dimethyl-3-propylimid- azolium iodide (PMII, Merk Ltd., Taiwan, Republic of China) 0.05 M I_2 _(Sigma-Aldrich China, Inc., Shanghai, People's Republic of China), and 0.5 M *tert*-butylpyridine (Sigma-Aldrich China, Inc., Shanghai, People's Republic of China) in acetonitrile.

The surface morphologies and dimensions of the ZnO/MWNT composite films were characterized using a FEI Nova230 FE-SEM (FEI Inc., Hillsboro, Oregon). TEM images of MWNTs were taken by employing a JEOL-JEM-1230 transmission electron microscope (Jeol (Beijing) Co., Ltd., Beijing, China). X-ray diffraction [XRD] patterns were obtained by using a diffractometer (PANalytical X'Pert PRO) (Spectris Instrumentation and Systems Shanghai Ltd., Shanghai, People's Republic of China) with Cu Kα radiation. The thickness of the ZnO nanocrystalline layer was measured by using a microfigure measuring instrument (Surfcorder ET3000, Kosaka Laboratory, Kosaka, Japan). The *η *was measured under a white light source (YSS-100A, Yamashita Denso Company, Tokyo, Japan), which gave an irradiance of 100 mW cm^-2 ^at the equivalent of air mass [AM] 1.5 on the surface of the solar cell. The irradiance of simulated light was calibrated using a silicon photodiode (BS-520, Bunko Keiki Co., Ltd, Tokyo, Japan). The evolution of the electron transport process in the cell was investigated using electrochemical impedance spectroscopy [EIS]. Impedance spectroscopy was performed using an electrochemical analyzer (Autolab PGSTAT30) (EcoChemie, Utrecht, The Netherlands). The impedance measurements were carried out by applying a direct current bias at open circuit voltage [*V*_OC_] and an alternating current voltage with amplitude of 10 mV in a frequency range from 10^-2 ^to 10^5 ^Hz under AM 1.5 illumination.

## Results and discussion

**Figure 1:**
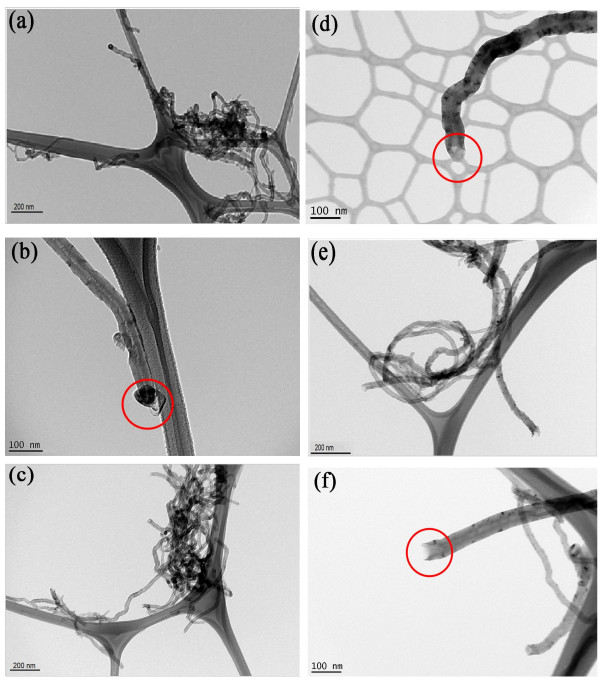
**TEM images of MWNTs**. (**a**) and (**b**) U-MWNTs; (**c**) and (**d**) O-MWNTs; (**e**) and (**f**) acid-MWNTs.

The TEM images of the pristine U-MWNT and the modified MWNTs (O-MWNT and acid-MWNT) are shown in Figure 1. As shown in Figure 1a, U-MWNTs exhibited highly curved, aggregated random-coil feature, which can be attributed to the intrinsic van der Waals attractions between the individual nanotubes. The black spot on the tip of U-MWNT (Figure 1b) was not observed on O-MWNT (Figure 1d), meaning that most of the impurities had been removed by air oxidation treatment. The air oxidation treatment purified the pristine MWNTs, but did not change significantly the aspect ratio or the aggregated random-coil nature of U-MWNTs, as demonstrated by Figure 1a, c. Figure 1e, f shows that the mixed acid treatment greatly modified the structure of MWNTs and opened the tip of acid-MWNTs. Figure 1e also shows that acid-MWNTs were no longer entangled together, which could be attributed to the carboxyl layer formed on the surface of acid-MWNTs. According to the Fourier transform infrared spectroscopy spectra reported earlier in the literature, the mixed acid treatment generates a carboxyl layer on the surface of CNTs [16]. However, the mix acid treatment also shortened MWNTs and generated significantly more defect sites on acid-MWNTs.

**Figure 2:**
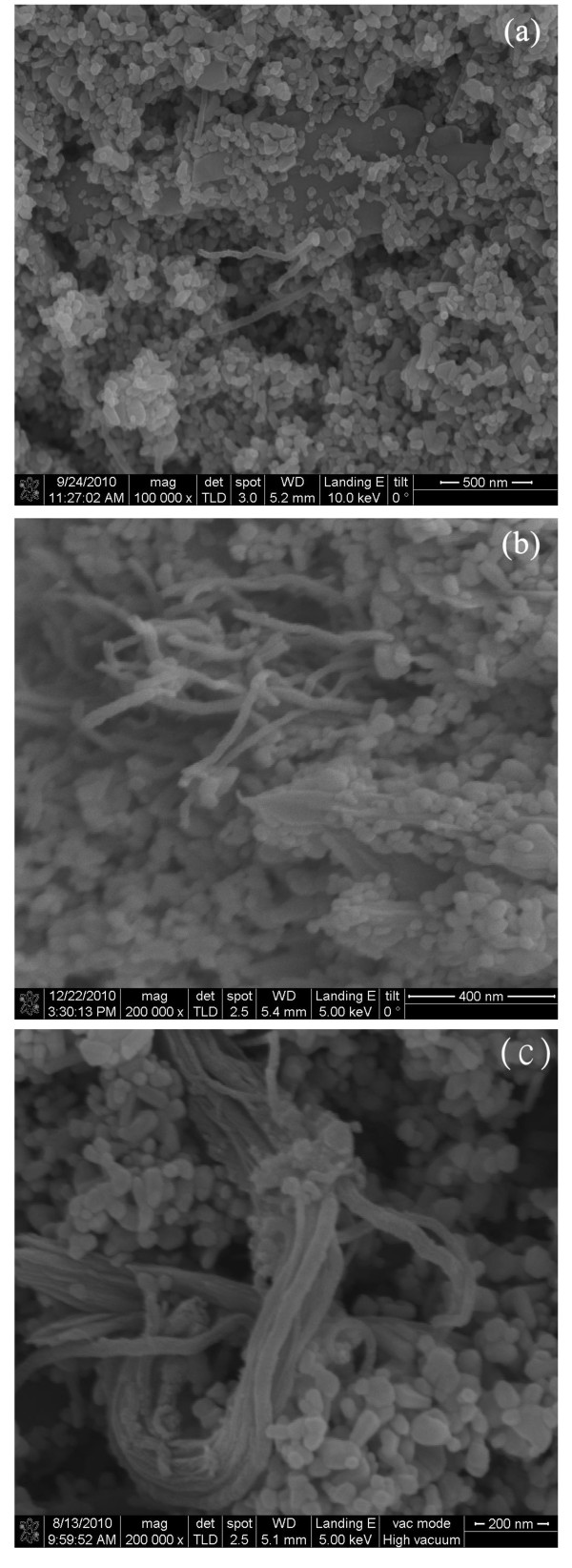
**FE-SEM micrographs of ZnO/MWNT composite film containing 0.01 wt% of MWNTs**. (**a**) top view and (**b**) cross-sectional view of ZnO/acid-MWNT film; (**c**) cross-sectional view of ZnO/O-MWNT film.

The FE-SEM micrograph in Figure 2a reveals the surface morphology of the ZnO/acid-MWNT film with exfoliated MWNTs visible on the surface. The cross-sectional view of the ZnO/acid-MWNT film, given in Figure 2b, shows that acid-MWNTs were embedded in the ZnO nanoparticle network. Although the acid-MWNTs were not uniformly distributed, they did not form bundles like O-MWNTs, as shown in Figure 2c. It is well known that the surface of ZnO particles possesses a lot of hydroxyl groups, which can undergo interaction with the carboxylic groups on acid-MWNT surface. This should be the reason why better dispersion was achieved with acid-MWNTs. The same principle has been applied in a previous study to reduce microcrack formation on electrophoretically deposited TiO_2 _film through the addition of acid-treated MWNTs [17]. Like ZnO, the surface of TiO_2 _particle also possesses hydroxyl groups. However, the mechanical mixing method used in this study was probably not sufficient to form uniformly dispersed ZnO/MWNT paste, so acid-MWNTs were not uniformly distributed in the resulting composite films.

**Figure 3:**
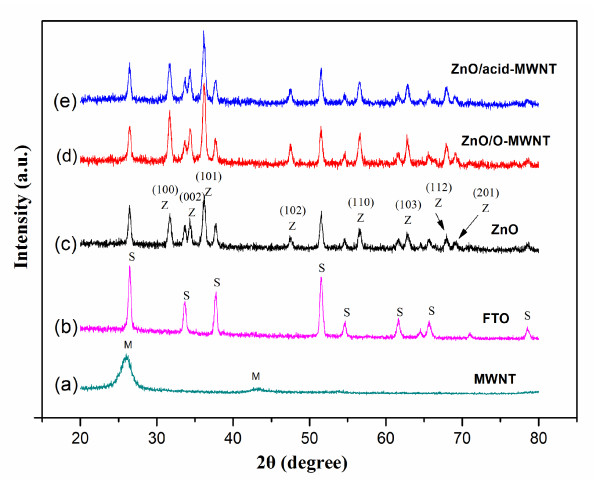
**XRD spectra**. (a) MWNT; (b) FTO glass substrate; (c) ZnO; (d) ZnO/O-MWNT film; (e) ZnO/acid-MWNT film. (M, MWNT; S, FTO substrate (SnO_2_); Z, ZnO).

The XRD spectra of the pure ZnO film and two ZnO/MWNT composite films are shown in Figure 3. All three films exhibited peaks at 2θ = 31.7°, 34.5°, 36.3°, 47.4°, 56.6°, 62.7°, and 68.2°, which correspond to the (100), (002), (101), (102), (110), (103), and (201) planes of ZnO (Joint Committee on Powder Diffraction Standards 36-1451), respectively [18]. However, the characteristic peak of MWNT (2θ = 26.3°) was not observed in the XRD spectra. The amount of MWNT in the composite films might be too small to generate visible peaks for MWNTs.

**Figure 4:**
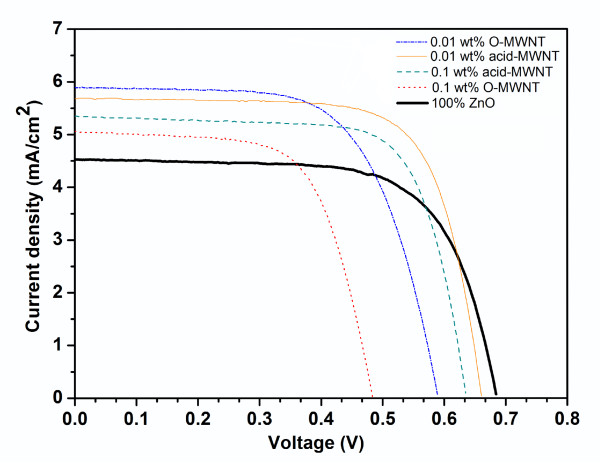
**J-V characteristics of fabricated DSSCs**.

**Figure 5:**
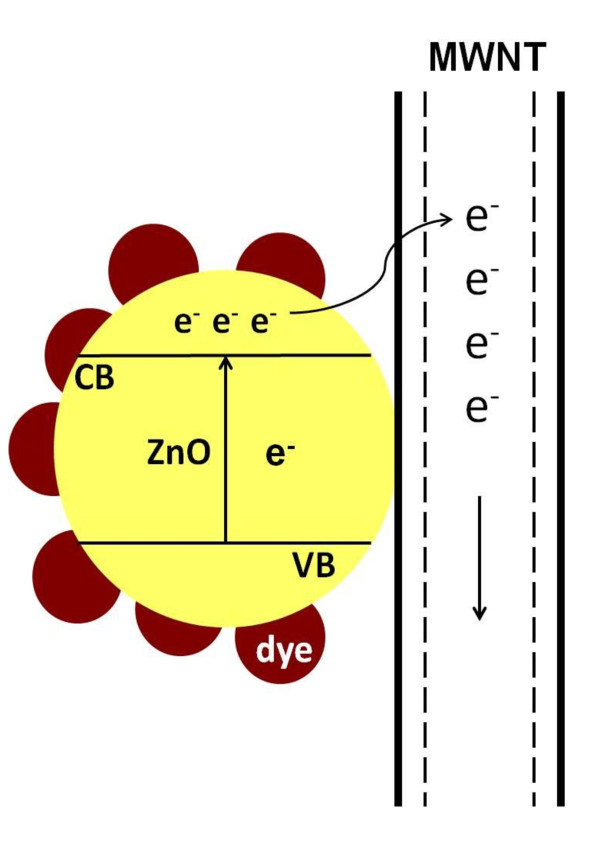
**Proposed mechanism for enhanced electron transport in ZnO/MWNT composite electrodes**. CB, conduction band; VB, valence band.

Comparison of the J-V characteristics of pure ZnO film-based and ZnO/MWNT composite film-based DSSCs is presented in Figure 4. The photoelectric conversion performances of the ZnO/MWNT film-based DSSCs depended strongly on the type and loading of MWNTs in the electrodes. The value of short-circuit photocurrent density [*J*_SC_] was greatly increased when 0.01 wt% of O-MWNTs or acid-MWNTs were incorporated in the electrode. It has been reported that the incorporation of a small amount of CNTs into the TiO_2 _films can enhance the collection and transport of electrons [11,12]. The DSSC containing 0.01 wt% of acid-MWNTs yielded the highest *η*. The peak *η *of 2.70% represents a 30% increase compared to that of the pure ZnO-based cell. The increase in *η *by the incorporation of 0.01 wt% of acid-MWNTs was attributed to a strong enhancement in *J*_SC _coupled with a slight decrease in *V*_OC_. The improvement of *η *for the DSSC modified by 0.01 wt% of acid-MWNTs was probably due to the decrease in the charge transport resistance at the ZnO/dye/electrolyte interface, a larger number of injected electrons, and more efficient electron transport effected by acid-MWNTs (see Figure 5). Lower *η *resulted when O-MWNT or a higher percentage of acid-MWNT was used. The decline in *η *was most significant in DSSC containing 0.1 wt% of O-MWNT, where the aggregation of MWNT was likely to be most severe. The degradation of DSSC performance was probably caused by a loss in optical transparency at higher MWNT loadings [10,11].

**Figure 6:**
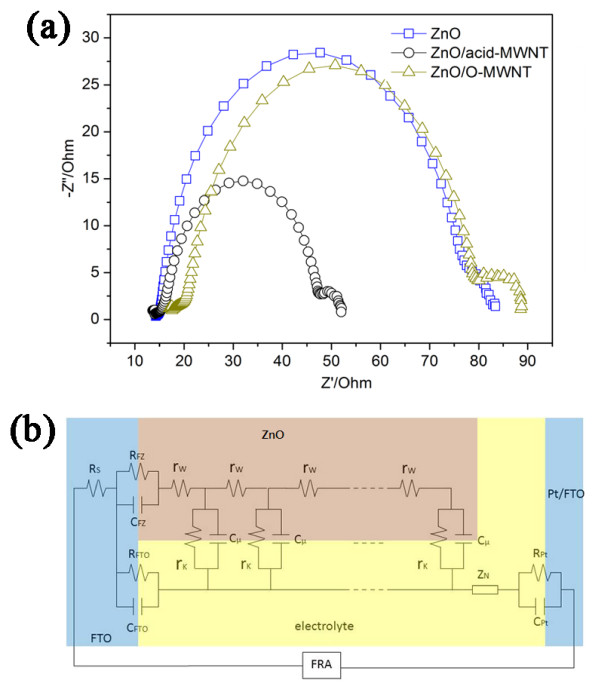
**Equivalent circuit and Nyquist plots**. (**a**) Equivalent circuit for the simulation of DSSC impedance spectra. (**b**) Nyquist plots of fabricated DSSCs determined under an AM 1.5 G solar illumination of 100 mW cm^-2^. MWNT loading was 0.01 wt%, and the film thickness was 8 μm.

To further understand the effect of MWNTs on cell performance, some of the solar cells were analyzed by EIS, and the experimental impedance data were fitted to an equivalent circuit. The experimental impedance data, obtained from DSSCs made with 8 μm-thick films, are given by the Nyquist plots in Figure 6a. The Nyquist plots in Figure 6a are the impedance spectra of the pure ZnO-based cell and the ones containing 0.01 wt% acid-MWNTs and 0.01 wt% O-MWNTs. The corresponding *J*_SC_, *V*_OC_, fill factor [FF], and conversion efficiencies of these cells are summarized in Table 1. Generally, the impedance spectra of DSSCs exhibit three semicircles. The semicircle in the high frequency range corresponds to charge transfer behavior at the Pt/electrolyte (*R*_Pt _and *C*_Pt_), the FTO/electrolyte (*R*_FTO _and *C*_FTO_) and the FTO/ZnO (*R*_FZ _and *C*_FZ_) interfaces. The semicircle in the middle frequency range is assigned to the electron transfer at the ZnO/dye/electrolyte interfaces, which is related to electron-transport resistance in the ZnO network [*R*_w_], charge-transfer resistance related to recombination of electrons at the ZnO/electrolyte interface [*R*_k_], and the chemical capacitance of the ZnO electrode [*C*_μ_]. The semicircle in the low frequency range reflects the Warburg diffusion process of I^-^/I_3_^- ^in the electrolyte (Z_N_) [19-21]. To extract DSSC parameters related to electron transport and recombination, the experimental impedance data was fitted to an equivalent circuit based on the diffusion-recombination model [19,20], as shown in Figure 6b. The results from fitting the Nyquist plot data to the equivalent circuit are summarized in Table 2. As shown, the incorporation of MWNTs lowered the *R*_w_. The lowest *R*_w _occurred when the photoelectrode was modified with 0.1 wt% of O-MWNTs. This was likely due to the high electrical conductivity of O-MWNTs. It should be noted that acid-MWNTs have considerably lower electrical conductivity than O-MWNTs [22], because mixed acid treatment leads to pronounced structural damages [15,22]. On the other hand, the incorporation of MWNTs also led to lower *R*_k_, which is related to the charge recombination between injected electrons and electron acceptors in the redox electrolyte at the ZnO/electrolyte interface, meaning recombination loss was more significant when photoelectrode was modified with MWNTs. This recombination loss was most severe with the O-MWNT-modified device, whose *R*_k _value was about half that of the pure ZnO-based cell. This was likely due to the severe aggregation of O-MWNT in the ZnO matrix. As shown in Figure 2c, O-MWNT formed bundles in the ZnO matrix. Therefore, only the outer layer of O-MWNTs was in contact with ZnO nanoparticles. The interior of the O-MWNT bundles was most likely exposed directly to electrolyte, resulting in the highest recombination loss. Despite their lower *R*_k _values, the MWNT-modified devices still had higher ratio of *R*_k _to *R*_w _[*R*_k_/*R*_w_] values than the pure ZnO device, suggesting that a larger number of electrons were injected in the MWNT-containing electrodes. This result could explain the higher *J*_SC _observed in MWNT-containing cells [20,23]. The reciprocal of the characteristic frequency (1/*ω*_max_) represents the mean electron lifetime [*τ*_eff_]. The *ω*_max _values were found to be 97.09, 145.98, and 125.01 Hz for the ZnO, ZnO/O-MWNT, and ZnO/acid-MWNT devices, respectively. The *τ*_eff _was longer in the pure ZnO-based device compared to the MWNT-containing devices (Table 2). This indicates that the incorporation of MWNTs increased the recombination reaction. This is consistent with the low *R*_k _values for MWNT containing cells. The effective electron diffusion coefficient in the photoanode [*D*_eff_] is calculated by using the relation: *D*_eff _= (*R*_k_/*R*_w_)(*L*^2^/*τ*_eff_), where *L *is the thickness of the ZnO film (8 μm). As shown in Table 2 the *D*_eff _for the MWNT-containing cells were much larger than that of the pure ZnO-based cell. The higher *D*_eff _for MWNT-modified DSSCs could be explained by more injected electrons and the induced faster transport of electrons. The effective electron diffusion length [*L*_eff_], calculated by the relation: *L*_eff _= (*D*_eff _× *τ*_eff_)^1/2^, expresses the competition between the collection and the recombination of electrons. The *L*_eff _values were estimated to be 35.4 μm for the ZnO/O-MWNT device and 27.1 μm for the ZnO/acid-MWNT device. The *L*_eff _in the MWNT-modified DSSCs was higher than that in the pure ZnO-based cells (22.2 μm). The higher value of *L*_eff _was consistent with the higher *J*_SC _values of the MWNT-modified cells (Table 1).

**Table 1:** Effects of incorporating MWNTs on J-V characteristics of DSSCs

Electrode composition	Short-circuit photocurrent density (mA/cm^2^)	Open circuit voltage (volt)	Fill factor	Energy conversion efficiency (percent)
**ZnO**	4.54	0.68	0.68	2.11
**ZnO/0.01 wt% O-MWNT**	5.90	0.60	0.66	2.33
**ZnO/0.01 wt% acid-MWNT**	5.68	0.66	0.72	2.70

The thickness of the ZnO or ZnO/MWNT film was 8 μm.

**Table 2:** Effects of incorporating MWNTs on the electron transport properties of fabricated cells

Electrode composition	Charge-transfer resistance at the ZnO/electrolyte interface [*R*_k_] (ohm)	Electron transport resistance in the ZnO network [*R*_w_] (ohm)	Ratio of *R*_k _to *R*_w _	Mean electron lifetime (ms)	Electron diffusion coefficient in the photoanode (10^-4 ^cm^2 ^s^-1^×)	Effective electron diffusion length (micrometer)
**ZnO**	50.17	6.49	7.73	10.30	4.80	22.2
**ZnO/0.01 wt% O-MWNT**	27.15	1.39	19.53	6.85	18.25	35.4
**ZnO/0.01 wt% acid-MWNT**	41.56	3.62	11.48	8.00	9.18	27.1

The thickness of the ZnO or ZnO/MWNT film was 8 μm.

As shown in Table 1 the ZnO/O-MWNT device had the highest *J*_SC _value, but its overall *η *was lower than that of the ZnO/acid-MWNT device. This was due to the low *V*_OC _value of the ZnO/O-MWNT device, which was likely resulted from the severe aggregation of O-MWNTs in the ZnO matrix. Because O-MWNTs formed bundles (Figure 2c), only the outer surface of the bundle was in contact with ZnO nanoparticles. The interior of the O-MWNT bundle was most likely exposed directly to electrolyte, resulting in the highest recombination loss (lowest *R*_k _as shown in Table 2) and consequently the lowest *V*_OC_. Therefore, despite its highest *J*_SC_, the ZnO/O-MWNT device had lower *η *than the ZnO/acid-MWNT cell.

## Conclusions

DSSCs based on ZnO/MWNT composite films were fabricated using a low-temperature, direct blending procedure and compared with the pure ZnO nanoparticle-based cells. The cell performance was found to depend on the loading and surface modification of MWNTs. At a loading of 0.01 wt%, both O-MWNT and acid-MWNT could enhance the *η *of DSSCs, which was attributed to higher *J*_SC _values. However, the acid-MWNT modified cell had higher *η*. This is because the acid-MWNTs were disentangled and better dispersed in the ZnO matrix, leading to less recombination loss as compared to the O-MWNTs containing cells. The solar cell containing 0.01 wt% of acid-MWNT yielded the highest *η*, which was characterized by the following parameters: *J*_SC _= 5.68 mA/cm^2^, *V*_OC _= 0.66 V, FF = 0.72, and *η *= 2.70%.

## Abbreviations

acid-MWNT: group-modified MWNT; CNT: carbon nanotube; DSSC: dye-sensitized solar cells; MWNTs: multiwalled carbon nanotubes; EIS: electrochemical impedance spectroscopy; FE-SEM: field emission scanning electron microscopy; FF: fill factor; FTO: fluorine-doped tin oxide; *J*_SC_: short-circuit photocurrent density; O-MWNT: oxidized MWNT; TEM: transmission electron microscopy; U-MWNT: unmodified-MWNT; *V*_OC_: open circuit voltage; XRD: X-ray diffraction.

## Competing interests

The authors declare that they have no competing interests.

## Authors' contributions

WCY proposed the idea, helped in the manuscript preparation, and oversaw the study. WCC designed and carried out the experiment, analyzed the data, and prepared the manuscript. YYC helped define the research theme and experimental design. YCY carried out the fabrication of ZnO/MWNT DSSCs. CHL provided a helpful discussion and helped in the manuscript preparation. HHK contributed to the preparation of MWNT. All authors read and approved the final manuscript.
